# Pharmacological TLR4 Activation Promotes Hepatocyte Proliferation and Liver Repair Through Coordinated IL-6 and TCDCA Signaling

**DOI:** 10.3390/biology15191725

**Published:** 2026-10-01

**Authors:** Xinlu Yu, Haoxin Ma, Chao Wang, Ke Feng, Pingxin Sun, Jiqianzhu Zhang, Junyu Lu, Hongxia Zhang, Chao Wang, Chunyan Wang, Haiying Zhu, Wenlin Li

**Affiliations:** 1 Department of Cell Biology, College of Basic Medical Science, Naval Medical University, Shanghai 200433, China; yuxinludy@163.com (X.Y.); vaa1234@163.com (H.M.); wangchao_smmu@163.com (C.W.); kefeng_bio@foxmail.com (K.F.); spxcellbiology@hotmail.com (P.S.); zjqztox@163.com (J.Z.); ljycloud@126.com (J.L.); zhhx_2006@aliyun.com (H.Z.); wangchaosmmu@126.com (C.W.); chywangnj@163.com (C.W.); 2 Department of Health Toxicology, Naval Medical University, Shanghai 200433, China; 3 Shanghai Key Laboratory of Cell Engineering, Naval Medical University, Shanghai 200433, China

**Keywords:** CRX-527, TLR4, JAK2/STAT3 signaling, hepatocyte proliferation, acute liver injury

## Abstract

The regenerative capacity of the liver is frequently insufficient following severe injury, and effective therapeutic strategies remain limited. Here, we identify CRX-527, a TLR4 agonist, as a novel inducer of hepatocyte proliferation in both healthy mice and a model of acetaminophen (APAP)-induced acute liver injury. Immunofluorescence analysis revealed that CRX-527-induced proliferative activity was predominantly observed in zone 2. Mechanistically, CRX-527 acts via TLR4 to induce transient IL-6 secretion, which subsequently activates the JAK2/STAT3 signaling pathway. In addition, metabolomic profiling identified taurochenodeoxycholic acid (TCDCA) as a synergistic partner of IL-6 in promoting hepatocyte proliferation. In APAP-injured mice, treatment with CRX-527 alone or in combination with IL-6 and TCDCA significantly reduced hepatic necrosis, accelerated tissue repair, and improved survival. Collectively, our findings uncover a previously unrecognized combined effect between IL-6/JAK2/STAT3 signaling and TCDCA in promoting hepatocyte proliferation and highlight CRX-527 and the IL-6/TCDCA combination as potential regenerative targets that warrant further preclinical evaluation for drug-induced liver injury, particularly in the setting of APAP overdose.

## Simple Summary

When the liver is severely damaged, it often cannot repair itself fully, and few treatments are available to help. In this study, we identified that clinically relevant TLR4 agonists, CRX-527 and MPLA, could stimulate the liver to regenerate. We gave CRX-527 or MPLA to healthy mice and to mice with liver injury caused by an overdose of a common painkiller (acetaminophen). We found that CRX-527 promoted hepatocyte division, particularly in zone 2 of the liver lobules. It worked by triggering a natural immune signal and by cooperating with a bile acid that the body produces naturally. Importantly, we also found that giving this immune signal together with the bile acid produced the same beneficial effects: it reduced liver damage, sped up healing, and improved survival in injured mice. These findings suggest that either CRX-527 alone, or a combination of the immune signal and the bile acid, could potentially offer more options for recovery after severe liver damage.

## 1. Introduction

The liver is the largest internal organ in the human body and orchestrates a wide range of essential physiological processes, including metabolism, detoxification, bile synthesis, and immune regulation. A defining feature of the liver is its remarkable capacity to regenerate following injury, a property critical for maintaining homeostasis [1,2]. However, in cases of severe or persistent damage, such as chronic viral hepatitis, alcohol-related liver disease, drug overdose, or major hepatectomy, this endogenous regenerative capacity can become overwhelmed, resulting in impaired liver function and, ultimately, life-threatening liver failure [3,4]. Although liver transplantation remains the gold standard treatment for end-stage liver disease, its clinical application is constrained by donor organ shortages, the risk of immune rejection, and high costs [5,6,7]. Consequently, there is an urgent need to develop novel therapeutic strategies that can safely and effectively harness the liver’s intrinsic regenerative potential.

Hepatocyte proliferation is orchestrated by multiple signaling pathways, including those activated by growth factors, cytokines and metabolic regulators [1,8]. Among these, the IL-6/JAK2/STAT3 axis is a well-established driver of liver regeneration that directly transduces proliferative signals to activate cell cycle-related genes [9,10,11]. In addition, emerging evidence suggests that bile acid metabolites can modulate hepatic gene expression programs involved in cell proliferation, lipid metabolism, and tissue repair [1,12]. Among the various bile acid species, taurochenodeoxycholic acid (TCDCA) has been shown to exert cytoprotective effects and promote hepatocyte survival under stress conditions [13]. Toll-like receptor 4 (TLR4) has been implicated in liver regeneration primarily through its role in sensing gut-derived lipopolysaccharide and inducing the production of pro-regenerative cytokines, including TNF-α and IL-6 [14]. However, whether direct pharmacological activation of TLR4 with a clinically relevant agonist is sufficient to drive robust hepatocyte proliferation in the quiescent adult liver remains unclear. Small-molecule compounds, owing to their structural diversity, ease of optimization, and capacity for precise pathway modulation, represent a promising approach for therapeutically targeting these regenerative circuits. Among these, CRX-527, a synthetic TLR4 agonist, provides a valuable tool for investigating whether direct TLR4 engagement can elicit a pro-proliferative response in hepatocytes.

Drug-induced liver injury (DILI) encompasses a spectrum of hepatic adverse events, ranging from mild transaminase elevation to acute liver failure. Here, we focus on intrinsic DILI caused by acetaminophen (APAP) overdose, a well-established model of acute hepatic necrosis driven by the reactive metabolite NAPQI following glutathione depletion [15,16,17,18]. This model provides a clinically relevant context in which to evaluate whether pharmacological activation of regenerative pathways can counteract extensive hepatocyte loss.

Therefore, the present study aimed to investigate the pro-proliferative efficacy of the TLR4 agonist CRX-527 in the liver, elucidate the underlying mechanisms, and determine whether this strategy confers protective effects against acute liver injury induced by APAP overdose. These findings may help clarify the mechanisms underlying CRX-527-induced liver regeneration and inform the development of novel therapeutic approaches for drug-induced liver injury.

## 2. Materials and Methods

### 2.1. Animal Experiment

Wild-type C57BL/6 mice (male, 6–10-week-old) were obtained from Shanghai Jihui Experimental Animal Breeding Co., Ltd., Shanghai, China (Production License No. SCXK [Shanghai] 2022-0009) and housed under controlled conditions (22–24 °C, 12 h light/dark cycle) with ad libitum access to food and water. All experimental procedures were approved by the Ethics Committee of Naval Medical University and conducted in accordance with the ARRIVE guidelines (Approval No: NMUMERC-2021-92168118, 10 August 2021). Mice were randomly assigned to experimental and control groups. Homozygous *Tlr4* knockout (*Tlr4*^−/−^) mice on a C57BL/6 background were kindly provided by Dr. Xinming Jia (Tongji University).

Unless otherwise specified, wild-type male mice aged 8 weeks were used in all experiments. All mice were ranked by body weight and randomly assigned to groups. All treatments were administered at approximately the same time of day. For in vivo treatments, mice received daily intraperitoneal injections for 7 consecutive days of the following compounds at the indicated doses: CRX-527 (0.25 mg/kg; InvivoGen, San Diego, CA, USA, cat. no. 216014-14-1), MPLA (0.5 mg/kg; Avanti Research, Alabaster, Alabama, USA, cat. no. 699800), resatorvid (5 mg/kg; MedChemExpress, Monmouth Junction, NJ, USA, cat. no. HY-11109), ruxolitinib (60 mg/kg; MedChemExpress, cat. no. HY-50858), 4-octyl itaconate (4-OI; 50 mg/kg; MedChemExpress, cat. no. HY-112675), tauroursodeoxycholic acid (TUDCA, 25 mg/kg; MedChemExpress, cat. no. HY-76847), taurochenodeoxycholic acid (TCDCA, 25 mg/kg; Selleck, Chemicals, Houston, TX, USA, cat. no. S3865,) or IL-6 (25 μg/kg; PeproTech, Cranbury, NJ, USA, cat. no. 216-16). Control mice received vehicle injections on the same schedule. At the end of the treatment period, mice were euthanized, and liver tissues and serum samples were collected for analysis.

For histological and immunofluorescence assessments (including necrotic area quantification and positive-cell counting), all tissue sections and microscopic images were coded with random numeric labels by an independent technician not involved in the analysis; decoding was performed only after all quantitative measurements were completed.

### 2.2. Acute Liver Failure (ALF) Model

For APAP-induced acute liver injury, 10–12-week-old wild-type C57BL/6 mice were fasted for 14 h prior to a single intraperitoneal injection of APAP (400 or 600 mg/kg; Sigma-Aldrich, St. Louis, MO, USA, cat. no. A7085). Two hours after APAP administration, mice received intraperitoneal injections of CRX-527, IL-6, TCDCA, IL-6 plus TCDCA, or vehicle. This time point was selected to allow the completion of APAP metabolic activation, enabling assessment of pro-regenerative efficacy independent of any interference with NAPQI formation. This design also reflects the clinically relevant scenario in which therapeutic intervention is initiated after the acute detoxification window has passed. Liver samples were collected at the indicated time points for histological and biochemical analysis.

Premature euthanasia criteria: (1) loss of righting reflex (inability to right itself within 30 s when placed supine) or unresponsiveness to tail-pinch; (2) severe generalized icterus accompanied by a >70% reduction in spontaneous activity lasting more than 6 h. Animals meeting any of these criteria were euthanized immediately, excluded from subsequent timepoint analyses, and recorded as idiosyncratic hyper-responders.

### 2.3. Hepatocytes Isolation and Primary Culture

Hepatocytes were isolated by a two-step collagenase perfusion method, as previously described [19,20]. Briefly, the liver was perfused with prewarmed (37 °C) liver perfusion buffer I for 5 min, followed by perfusion buffer II (DMEM/F12 (Gibco, Thermo Fisher Scientific, Waltham, MA, USA, cat. no. 11320033) supplemented with 10 mM HEPES (Gibco,cat. no. 15630130) and 0.5 mg/mL collagenase IV (Roche, Basel, Switzerland, cat. no. 11088858001)) for approximately 15 min. The liver was then excised and incubated in buffer II at 37 °C for an additional 10 min. The tissue was gently dissociated, and the cell suspension was filtered through a 70 μm cell strainer (Falcon, Corning, Corning, NY, USA, cat. no. 352350), followed by centrifugation at 100× *g* for 1 min at 4 °C. Cell viability was determined by Trypan blue exclusion, and only preparations with >85% viability were used. Experiments were performed using hepatocytes from at least three independent isolations, each from a distinct mouse. The hepatocyte-enriched pellet was used for bulk RNA-seq or primary culture.

For primary culture, plates were pre-coated with 1% Matrigel (growth factor-reduced, Corning, cat. no.356231) diluted in DMEM/F12 and incubated at 4 °C overnight. Freshly isolated hepatocytes were seeded at 1 × 10^5^ cells/mL in basal medium consisting of DMEM/F12 supplemented with 0.5× N2(Gibco, cat. no.17502048), 0.5× B27(Gibco, cat. no. 17504044), 2 mM nicotinamide (Sigma-Aldrich, cat. no. N0636), 100 nM dexamethasone (Sigma, cat. no. D4902), 60 μg mL^−1^ 2-phospho-L-ascorbic acid (Sigma, cat. no. A8960), and 1% penicillin/streptomycin (Gibco, cat. no. 15140122). The medium was replaced after 30 min to remove non-adherent cells. For treatment experiments, cells were exposed to IL-6 (20 ng/mL), TCDCA (100 μM), or IL-6 plus TCDCA. Culture medium was refreshed daily.

### 2.4. RNA-Sequencing Analysis

Total RNA was extracted using the TRIzol reagent (Invitrogen, Carlsbad, CA, USA, cat. no.15596026CN). RNA integrity was assessed using an Agilent 2100 Bioanalyzer (Agilent Technologies, Santa Clara, CA, USA). Libraries were constructed using VAHTS Universal V6 RNA-seq Library Prep Kit (Vazyme, Nanjing, China, cat. no.NR616) according to the manufacturer’s instructions and sequenced on an Illumina NovaSeq 6000 platform (Illumina, San Diego, CA, USA). Raw reads in FASTQ format were preprocessed using fastp (v 0.20.1) to remove low-quality reads and adaptor sequences. Clean reads were aligned to the reference genome using HISAT2 (v 2.1.0). Gene expression levels (FPKM) and raw read counts were quantified using HTSeq-count (v 0.11.2). Differentially expressed genes were identified using DESeq2 (v 1.22.2), with significance thresholds set at *p* < 0.05 and fold change > 2 or < 0.5.

### 2.5. Tissue Processing and Immunocytochemistry

Liver tissues were embedded in OCT compound and stored at −80 °C. Sections (5 μm) were prepared using a cryostat set at −20 °C and mounted onto adhesion slides. For immunofluorescence staining, sections were fixed in 4% paraformaldehyde for 5 min at room temperature, washed three times with PBST (PBS containing 0.2% Triton X-100), and blocked with 5% normal donkey serum for 30 min. Sections were then incubated with primary antibodies overnight at 4 °C. After washing, sections were incubated with fluorochrome-conjugated secondary antibodies for 30 min at room temperature, followed by nuclear counterstaining with DAPI staining (1:1000 dilution) for 5 min. Images were acquired using a Nikon Eclipse 50i fluorescence microscope (Nikon Corporation, Tokyo, Japan).

For zonal analysis of hepatocyte proliferation, hepatic lobules were divided into three zones based on immunofluorescence staining for zone-specific markers: zone 1 (periportal) was identified by E-cadherin positivity, zone 3 (pericentral) by glutamine synthetase (GS) positivity, and zone 2 (midlobular) was defined as the intervening region negative for both markers. Ki67-positive hepatocytes were manually counted and assigned to each zone accordingly. For each animal, three randomly selected microscopic fields (×200 magnification) were evaluated. Data are presented as the absolute distribution of Ki67-positive cells across the three zones.

### 2.6. IL-6 ELISA

Serum IL-6 concentrations were measured using a mouse IL-6 OneStep ELISA kit (STARTER, Hangzhou, China, cat. no. S0C3019) according to the manufacturer’s instructions. Briefly, standards and samples (50 μL/well) were added in duplicate to pre-coated plates, followed by 50 μL of antibody cocktail. Plates were incubated at 25 °C for 45 min with orbital shaking at 500 rpm. After washing, 100 μL TMB substrate was added and incubated for 10 min in the dark. The reaction was stopped by addition of 100 μL stop solution, and absorbance was measured at 450 nm.

### 2.7. Metabolite Analyses

Serum samples were collected and immediately stored at −80 °C until analysis. For sample preparation, aliquots of 100 µL serum were thawed on ice, and proteins were precipitated by adding 400 µL of methanol–acetonitrile (V:V = 2:1) containing mixed internal standards (4 µg/mL). The mixture was vortexed for 1 min, followed by ultrasonic extraction in an ice-water bath for 10 min, and then stored at −40 °C overnight. After centrifugation at 12,000 rpm for 20 min at 4 °C, 150 µL of the supernatant was transferred into LC–MS vials with glass inserts for analysis.

Untargeted metabolomic profiling was performed using an LC-MS/MS system consisting of a Waters Acquity UPLC I-Class Plus and Orbitrap Q ExactiveTM HF mass spectrometer (Thermo Fisher Scientific). The analytical conditions were as follows: UPLC: column, Acquity UPLC HSS T3 (1.8 μm, 2.1 mm × 100 mm); column temperature, 45 °C; flow rate, 0.35 mL/min; injection volume, 5 μL; solvent system, water (0.1% formic acid): acetonitrile; gradient program, 95:5 *v*/*v* at 0 min, 95:5 *v*/*v* at 2 min, 70:30 *v*/*v* at 4 min, 50:50 *v*/*v* at 8 min, 20:80 *v*/*v* at 10.0 min, 0:100 *v*/*v* at 14 min, 0:100 *v*/*v* at 15 min 95:5 *v*/*v* at 15.1 min, 95:5 *v*/*v* at 16.0 min; MS: ion source, HESI; ion mode, positive and negative; scan range, *m*/*z* 70–1050; resolution, 60,000 (full scan) and 15,000 (MS/MS).

Raw data were processed using XCMS v4.5.1 software for baseline filtering, peak detection, integration, and retention time alignment. Ion peaks with RSD > 0.3 were removed, and remaining zero values were replaced with half of the minimum intensity across all samples, followed by log2 transformation. The qualitative score (maximum 80) is composed of four components: precursor mass accuracy (20 points), MS/MS fragmentation matching (20 points), isotope distribution matching (20 points), and retention time matching (20 points); compounds with a score < 36 were excluded. Annotation confidence was assigned as Level 1 (RT ± 0.3 min and Fragmentation Score ≥ 45), Level 2 (RT ± 0.3 min and Score < 45), Level 3 (Score ≥ 45 without RT), or Level 4 (Score < 45 without RT); metabolites at Level 1 was prioritized for validation. Differentially abundant metabolites were selected based on *p* < 0.05 and FC ≥ 2.0 or FC ≤ 1/2.0.

Principal component analysis (PCA) was performed to evaluate overall sample distribution and analytical reproducibility. Supervised partial least squares-discriminant analysis (PLS-DA) and orthogonal PLS-DA (OPLS-DA) were subsequently applied to distinguish metabolic profiles between groups and identify differentially abundant metabolites.

### 2.8. Statistical Analysis

Data are presented as the mean ± standard deviation (SD). Statistical analyses were performed using GraphPad Prism 8.0 software. Comparisons between two groups were performed with two-tailed unpaired Student’s *t*-test. For multiple-group comparisons, one-way analysis of variance (ANOVA) was performed, followed by Tukey’s post hoc test for multiple pairwise comparisons. For two-factor interactive designs, two-way ANOVA followed by Sidak’s post hoc correction. Survival curves were generated using the Kaplan–Meier method and compared by the log-rank test. Significance levels are indicated as follows: ns, not significant (*p* > 0.05); * *p < 0.05*; ** *p* < 0.01; *** *p* < 0.001; **** *p* < 0.0001. All quantitative experiments were performed with at least three independent biological replicates. All animals and data points were included.

For the CRX-527-induced hepatocyte proliferation assay, we performed an a priori power analysis (α = 0.05, power = 0.8) based on pre-experimental data and previous experimental experience from our research group. The analysis indicated that only 2 mice/group were theoretically sufficient.

For the CRX-527 survival experiment following APAP overdose, sample sizes were guided by an a priori power analysis based on pilot data; however, the final group sizes (vehicle, *n* = 6; CRX-527, *n* = 7) did not fully reach the calculated target due to practical constraints encountered during the study. This sample-size shortfall is acknowledged as a limitation in the Discussion. For the IL-6/TCDCA survival experiment following APAP overdose, no formal a priori power calculation was performed; final cohort sizes were 11–12 mice per group.

## 3. Results

### 3.1. CRX-527 Induces Hepatocyte Proliferation

To determine whether TLR4 activation is sufficient to promote liver regeneration, male C57BL/6 mice received daily intraperitoneal injections of CRX-527 for 7 days (Figure 1A). CRX-527 administration resulted in a significant increase in both liver weight and the liver-to-body weight ratio (Figure 1B,C). Hepatocyte proliferative activity was assessed by immunofluorescence staining for Ki67 and phospho-histone H3 (pHH3), which revealed a marked increase in Ki67-positive cells following CRX-527 treatment (Figure 1D,E). Hematoxylin and eosin (H&E) staining showed no evidence of liver injury after CRX-527 treatment (Supplementary Figure S1A). Consistently, serum levels of alanine aminotransferase (ALT), aspartate transaminase (AST), total bilirubin (T-BIL), and alkaline phosphatase (ALP) were comparable between vehicle- and CRX-527-treated groups (Supplementary Figure S1B–E). These findings indicate that CRX-527-induced hepatocyte proliferation occurs in the absence of overt liver injury. In addition, CRX-527 treatment upregulated hepatic mRNA expression of proliferation-related genes and TLR4 target genes, consistent with an association between TLR4 activation and hepatocyte proliferation (Figure 1F).

### 3.2. The Pro-Proliferative Effect of CRX-527 Is Dependent on TLR4

To confirm that the pro-proliferative effect of CRX-527 is mediated specifically through TLR4 rather than off-target effects, we evaluated a structurally distinct TLR4 agonist, monophosphoryl lipid A (MPLA), which is approved clinically as a vaccine adjuvant. In WT mice, treatment with MPLA for 7 days did not significantly increase the liver-to-body weight ratio (Figure 2A), but significantly elevated the proportion of Ki67-positive cells (Figure 2B,C). This proliferative response occurred in the absence of hepatocellular injury, as indicated by normal histology and unchanged serum liver function markers (Supplementary Figure S2A–E). To further confirm the specificity of TLR4 signaling, we employed the selective TLR4 antagonist resatorvid. Resatorvid significantly suppressed CRX-527- and MPLA-induced hepatocyte proliferation (Figure 2A–C).

To directly test TLR4 dependence, we compared the response to CRX-527 in WT and *Tlr4^−/−^* mice treated with vehicle or CRX-527 for 7 days (Figure 2D). CRX-527 significantly increased the liver-to-body weight ratio in WT mice relative to vehicle-treated group, whereas this effect was completely abolished in *Tlr4^−/−^* mice (Figure 2E). Accordingly, immunofluorescence staining for Ki67 and pHH3 revealed abundant proliferating hepatocytes in CRX-527-treated WT mice, but not in *Tlr4^−/−^* mice (Figure 2F,G). Together, these results establish that the pro-proliferative effect of CRX-527 is strictly dependent on TLR4.

### 3.3. CRX-527 Preferentially Promotes Hepatocyte Proliferation in Zone 2 and Activates Cell Cycle Programs

To characterize the spatial distribution of CRX-527-induced proliferation, we performed co-immunofluorescence staining for the zone 1 marker E-cadherin, the zone 3 marker glutamine synthetase (GS), and Ki67. Following CRX-527 or MPLA treatment, Ki67-positive cells were predominantly localized to zone 2 (Figure 3A). Quantitative analysis confirmed that approximately 49% of proliferating hepatocytes resided in zone 2 after CRX-527 treatment, significantly more than in zones 1 and 3 (Figure 3B). RNA-sequencing (RNA-seq) of hepatocytes revealed that CRX-527 significantly upregulated a core set of proliferation-associated genes, including *Ccna2*, *Ccnb2*, *Cdc20*, *Cdk1*, and *Mki67* in hepatocytes (Figure 3C). Gene Ontology (GO) enrichment analysis further demonstrated significant enrichment of pathways related to cell cycle progression, mitosis, and chromosome segregation (Figure 3D). Immunofluorescence co-staining for Ki67 with hepatocyte markers HNF4A or ALB confirmed that a substantial proportion of proliferating cells were hepatocytes (Supplementary Figure S3A–C). Collectively, these data indicate that CRX-527 activates cell cycle transcriptional programs and preferentially promotes hepatocyte proliferation in zone 2.

### 3.4. CRX-527-Induced Hepatocyte Proliferation Requires IL-6/JAK2/STAT3 Signaling

As TLR4 activation can induce downstream cytokines, including TNF-α and IL-6, both of which play a role in liver regeneration [21,22], we hypothesized that CRX-527 induces hepatocyte proliferation via these mediators. RNA-seq analysis confirmed that CRX-527 significantly upregulated hepatic expression of *Tnf* and *Il-6* (Figure 4A,B). To determine the relative contribution of each cytokine, we administered CRX-527 in combination with either an IL-6 neutralizing antibody or the TNF-α inhibitor adalimumab (ADA). IL-6 neutralization markedly reduced the CRX-527-induced increase in liver-to-body weight ratio, whereas ADA had no inhibitory effect (Figure 4C,D). Consistently, Ki67 staining demonstrated that IL-6 blockade, but not TNF-α inhibition, decreased CRX-527-induced hepatocyte proliferation (Figure 4E,F). These findings establish that CRX-527-driven hepatocyte proliferation requires IL-6 signaling.

To characterize the kinetics of CRX-527-induced IL-6 secretion in vivo, we measured serum IL-6 levels following a single administration. IL-6 peaked at 2 h post-injection and returned to baseline by 6 h, indicating a transient secretory profile (Figure 4G). Repeated daily administration over 7 days did not attenuate this response; serum IL-6 levels remained elevated at 2 h post-dose across all time points examined (Figure 4H). Together, these results demonstrate that CRX-527 induces transient IL-6 secretion, which is required for hepatocyte proliferation.

Because IL-6 canonically signals through the JAK1/2/STAT3 pathway to regulate hepatocyte cell cycle entry [23,24,25], we examined whether this cascade mediates CRX-527-induced proliferation. Using the selective JAK1/2 inhibitor ruxolitinib, which blocks STAT3 phosphorylation by competitively occupying the ATP-binding site of JAK1/2 kinases [26,27,28], we co-treated mice with ruxolitinib or vehicle alongside daily CRX-527 for 7 days. Although the liver-to-body weight ratio showed a slight, non-significant trend toward increase in the ruxolitinib plus CRX-527 group relative to vehicle controls (Supplementary Figure S5A), immunofluorescence staining for Ki67 and pHH3 revealed abundant proliferating hepatocytes in the CRX-527-treated group, whereas only sparse positive cells were observed in both the CRX-527 plus ruxolitinib and vehicle groups (Supplementary Figure S5B). Quantitative analysis confirmed that ruxolitinib substantially reduced the proportion of Ki67-positive hepatocytes (Supplementary Figure S5C). Western blot results showed that CRX-527 treatment robustly induced STAT3 phosphorylation in mouse livers, while co-administration of JAK1/2 inhibitor completely abolished this effect (Figure 4I). These results demonstrate that the JAK1/2/STAT3 pathway is essential for CRX-527-induced hepatocyte proliferation.

### 3.5. TCDCA Exerts a Combined Effect with IL-6 to Promote Hepatocyte Proliferation

When IL-6 was neutralized, the liver-to-body weight ratio in the CRX-527-treated group remained significantly higher than in the vehicle group (Figure 4D). Similarly, co-treatment with ruxolitinib did not fully normalize the liver-to-body weight ratio to vehicle levels (Supplementary Figure S5A), suggesting the existence of IL-6-independent pro-proliferative mechanisms downstream of CRX-527. Given the emerging role of metabolites as active signaling molecules capable of regulating liver regeneration through specific receptors [11,13], we hypothesized that CRX-527-induced changes in the metabolome might contribute to hepatocyte proliferation. To test this, we performed untargeted LC-MS/MS-based metabolomic profiling of serum from vehicle- and CRX-527-treated mice, which revealed clear separation between groups (Supplementary Figure S6A). Among 3144 detected metabolites, 139 were significantly upregulated and 559 were downregulated (Supplementary Figure S6B–D). Among these, CRX-527 treatment was accompanied by increased levels of twelve high-confidence (Level 1) metabolites, including itaconic acid (ITA), tauroursodeoxycholic acid (TUDCA), and taurochenodeoxycholic acid (TCDCA) (Figure 5A).

To assess whether these elevated metabolites contribute to hepatocyte proliferation, we first evaluated the cell membrane permeable itaconate derivative 4-octyl itaconate (4-OI) in vivo. Immunofluorescence staining revealed no significant difference in the proportion of Ki67-positive cells between 4-OI-treated and vehicle-treated mice (Supplementary Figure S6E,F), indicating that 4-OI does not promote hepatocyte proliferation under physiological conditions.

We next examined the effects of TUDCA and TCDCA. Male C57BL/6 mice were administered 25 mg/kg TUDCA or TCDCA daily for 7 days. TUDCA had no effect on the liver-to-body weight ratio, whereas TCDCA significantly increased this parameter (Figure 5B). Consistently, immunofluorescence staining revealed a marked increase in Ki67- and pHH3-positive hepatocytes in TCDCA-treated mice, whereas TUDCA-treated mice showed levels comparable to controls (Figure 5C,D). These results identify TCDCA as a bile acid metabolite capable of promoting hepatocyte proliferation in the uninjured liver.

Given that CRX-527 triggers IL-6 secretion and elevates circulating TCDCA levels, and that both factors independently promote hepatocyte proliferation, we hypothesized that they have a combined effect. To test this, mice were treated with PBS, IL-6 alone, TCDCA alone, or their combination. Notably, the combination treatment resulted in a substantially greater number of Ki67- and pHH3-positive cells than either monotherapy (Figure 5E). Quantitative analysis showed that exogenous IL-6 alone did not significantly increase the proportion of Ki67-positive hepatocytes, whereas TCDCA alone induced a moderate increase; importantly, co-administration of IL-6 and TCDCA significantly enhanced hepatocyte proliferation beyond the effect of either agent alone (Figure 5F). This synergistic effect was further confirmed in vitro, primary hepatocytes co-treated with IL-6 and TCDCA exhibited a significantly higher proportion of Ki67-positive cells than cells treated with either factor alone (Figure 5G,H). Collectively, these data demonstrate that exogenous IL-6 is insufficient to drive hepatocyte proliferation in isolation but can act in concert with TCDCA to elicit a robust proliferative response.

### 3.6. CRX-527 or IL-6/TCDCA Treatment Attenuates APAP-Induced Acute Liver Injury

Acetaminophen (APAP) overdose is the leading cause of acute liver failure in developed countries, and a single high-dose APAP administration in mice is a well-validated experimental model of this condition [1,16]. To evaluate the therapeutic potential of CRX-527, mice received a lethal dose of APAP (600 mg/kg), followed 2 h later by CRX-527 administration. CRX-527 treatment significantly improved survival relative to vehicle controls (Figure 6A). Because the 600 mg/kg dose resulted in high mortality in control animals, precluding detailed histological analysis, subsequent experiments used a sublethal dose of 400 mg/kg APAP. Histological analysis revealed markedly reduced necrotic areas at 24 and 48 h and accelerated tissue repair in CRX-527-treated mice compared with vehicle controls (Figure 6B,C). Serum ALT and AST levels were significantly lower in the CRX-527-treated group at 24 h (Figure 6D,E), further supporting a hepatoprotective effect.

APAP is primarily metabolized by CYP2E1 in pericentral (zone 3) hepatocytes, resulting in localized centrilobular necrosis. Immunostaining for the periportal marker E-cadherin and the pericentral marker CYP2E1 showed that pericentral injury peaked at 48 h post-APAP, at which point only one to two layers of CYP2E1-positive hepatocytes remained at the necrotic boundary. In contrast, CRX-527-treated mice exhibited markedly more rapid restoration of CYP2E1-positive hepatocytes in the pericentral zone (Figure 6F,G), indicative of accelerated regeneration of the injured compartment.

To determine whether the synergistic pro-proliferative effect of IL-6 and TCDCA observed in healthy mice could be translated to a therapeutic context, we evaluated this combination in the APAP model. Co-administration of IL-6 and TCDCA significantly improved survival following a lethal 600 mg/kg APAP challenge (Figure 7A). In the sublethal APAP model (400 mg/kg), histological analysis demonstrated that the combination treatment markedly reduced necrotic area at 48 h compared with PBS or IL-6 alone, although all groups exhibited extensive centrilobular necrosis at earlier time points (Figure 7B,C). Serum biochemical analysis revealed that IL-6 monotherapy paradoxically worsened liver injury at early time points, and this elevation likely reflects the pro-inflammatory effect of sustained IL-6 signaling in the absence of additional cytoprotective factors. However, TCDCA co-administration with IL-6 conferred significant hepatoprotection, as reflected by substantially lower ALT and AST levels at 48 h, supporting a combined effect wherein TCDCA complements IL-6 signaling to promote tissue repair (Figure 7D,E).

CYP2E1 immunostaining showed that TCDCA administered alone or in combination with IL-6, accelerated early recovery of CYP2E1-positive hepatocytes in the pericentral zone, whereas IL-6 alone had no beneficial effect. By 120 h, pericentral zone recovery was comparable across all groups (Figure 7F,G). Taken together, these findings further support a combined effect between IL-6 and TCDCA in promoting liver repair and identify this combination as a potential regenerative strategy that warrants further preclinical investigation in murine models of acute liver injury.

## 4. Discussion

Although TLR4 has long been implicated in liver regeneration through cytokine-mediated mechanisms [14,29], whether its direct pharmacological activation is sufficient to drive hepatocyte proliferation in vivo had remained unresolved. Here, we demonstrate that the clinically relevant TLR4 agonist CRX-527 or MPLA significantly promotes hepatocyte proliferation in vivo. This effect was validated using *Tlr4* knockout mice. Importantly, the pro-proliferative effects of both CRX-527 and MPLA occurred without inducing overt hepatocellular injury, suggesting that therapeutic TLR4 agonism can engage regenerative programs selectively.

An intriguing finding of our study is the spatial restriction of CRX-527-induced proliferation to the midzonal (zone 2) region. Although proliferating hepatocytes were observed across all zones, their significant enrichment in zone 2 suggests that midzonal hepatocytes possess distinct properties that render them particularly responsive to TLR4-mediated regenerative signals. Recent studies have proposed zone 2 hepatocytes as a key source for homeostatic liver renewal, contributing to parenchymal maintenance under physiological condition [30,31,32]. Our findings extend this concept by demonstrating that this population can be pharmacologically recruited to enhance post-injury regeneration. Whether this spatial preference reflects differential expression of TLR4 pathway components, heightened responsiveness to IL-6, or metabolic characteristics of zone 2 hepatocytes remains to be determined. Elucidating the molecular basis of this regional sensitivity may enable the development of more precisely targeted regenerative therapies.

An unexpected finding from our metabolomic analysis was the identification of TCDCA as a pro-proliferative metabolite. The observation that TCDCA alone promotes hepatocyte proliferation and, more importantly, exerts a cooperative effect with IL-6, adds a new dimension to our understanding of the regenerative microenvironment. Bile acids are increasingly recognized as signaling molecules beyond their classical roles in lipid digestion and cholesterol homeostasis [1,12,13,33]. As a taurine-conjugated bile acid, TCDCA may signal through receptors such as TGR5 or FXR [34,35,36,37,38], although the receptor mediating its pro-proliferative effect in hepatocytes remains to be identified. Mechanistically, TCDCA binding to TGR5 activates the intracellular cAMP–PKA–CREB axis [39], which directly promotes pro-survival gene expression while suppressing NF-κB activity and reducing pro-inflammatory cytokine production. In addition, the related bile acid CDCA has been shown to activate AMPK and subsequently ERK1/2 through FXR-dependent mechanisms [13], suggesting that TCDCA may engage a parallel mitogenic pathway via FXR/ERK signaling. Beyond these direct proliferative signals, TCDCA has also been reported to inhibit hepatocyte pyroptosis, thereby attenuating DAMP release and interrupting the inflammatory–necrotic cycle [40]. Collectively, these findings suggest that TCDCA promotes liver repair through a multi-pronged mechanism combining direct proliferative signaling with indirect anti-inflammatory effects, which together may have a combined effect with IL-6-driven regeneration. Notably, exogenous IL-6 alone is insufficient to drive robust hepatocyte proliferation, whereas its combination with TCDCA elicits a markedly stronger proliferative response. This combined effect represents a key mechanistic advance of the present study and has clear therapeutic implications, as the IL-6/TCDCA combination outperforms monotherapy in promoting recovery from APAP-induced liver injury.

Acetaminophen overdose remains a leading cause of acute liver failure, with treatment options currently limited to N-acetylcysteine (NAC) and supportive care [15,16,41,42,43,44]. NAC is most effective when administered early, acting primarily by replenishing glutathione stores to neutralize the reactive metabolite NAPQI, but it does not directly promote hepatocyte regeneration once injury is established. Our study demonstrates that CRX-527 monotherapy significantly reduces hepatic necrosis, accelerates histological repair, and improves survival in a clinically relevant APAP overdose model by actively promoting the proliferation of surviving hepatocytes to replace necrotic tissue. The IL-6/TCDCA combination represents an alternative regenerative strategy with comparable therapeutic efficacy. Together, these approaches have the potential to complement existing therapies by extending the actionable therapeutic window beyond the early detoxification phase.

Although we have provided a mechanistic framework for CRX-527 in promoting liver regeneration and discussed its potential applicability to APAP-induced injury, this study still has several limitations. First, although we provide a clear rationale for the predominant use of male mice, namely to avoid confounding effects of the estrous cycle on hepatic metabolism and regeneration, the exclusive use of a single sex limits the generalizability of our findings, and future studies in female mice are warranted. The survival experiments were conducted with relatively small cohorts. For the CRX-527 experiment, the final sample size (vehicle: *n* = 6; CRX-527: *n* = 7) fell short of the minimum indicated by our own a priori power calculation based on the day-7 endpoint (*n* = 9 per group); for the IL-6/TCDCA experiment, group sizes (*n* = 11–12 per group) were not derived from a prospective power calculation. These survival findings should therefore be interpreted with appropriate caution, and independent replication in adequately powered cohorts is warranted to confirm them. Second, our study relied solely on the APAP model, a classic paradigm of intrinsic DILI, raising the question of broader applicability. The IL-6/JAK2/STAT3 axis is also activated in ischemia-reperfusion injury and viral hepatitis, suggesting that our findings may extend beyond APAP-induced injury. In idiosyncratic DILI, however, immunoallergic mechanisms predominate, and TLR4/IL-6 stimulation could paradoxically exacerbate injury. Thus, although our findings offer proof of concept, validation across diverse models is required. Third, although we propose a mechanistic framework for TCDCA action involving potential FXR/TGR5-mediated pathways, we have not experimentally identified the receptor or downstream signaling cascade responsible for its pro-proliferative effect.

## 5. Conclusions

In conclusion, the present study identifies a previously unrecognized interaction between IL-6/JAK2/STAT3 signaling and TCDCA in promoting hepatocyte proliferation, with CRX-527-induced proliferative activity predominantly observed in zone 2 of the healthy liver. Both CRX-527 monotherapy and the IL-6/TCDCA combination represent candidate regenerative targets that warrant further preclinical investigation for the treatment of DILI.

## Figures and Tables

**Figure 1:**
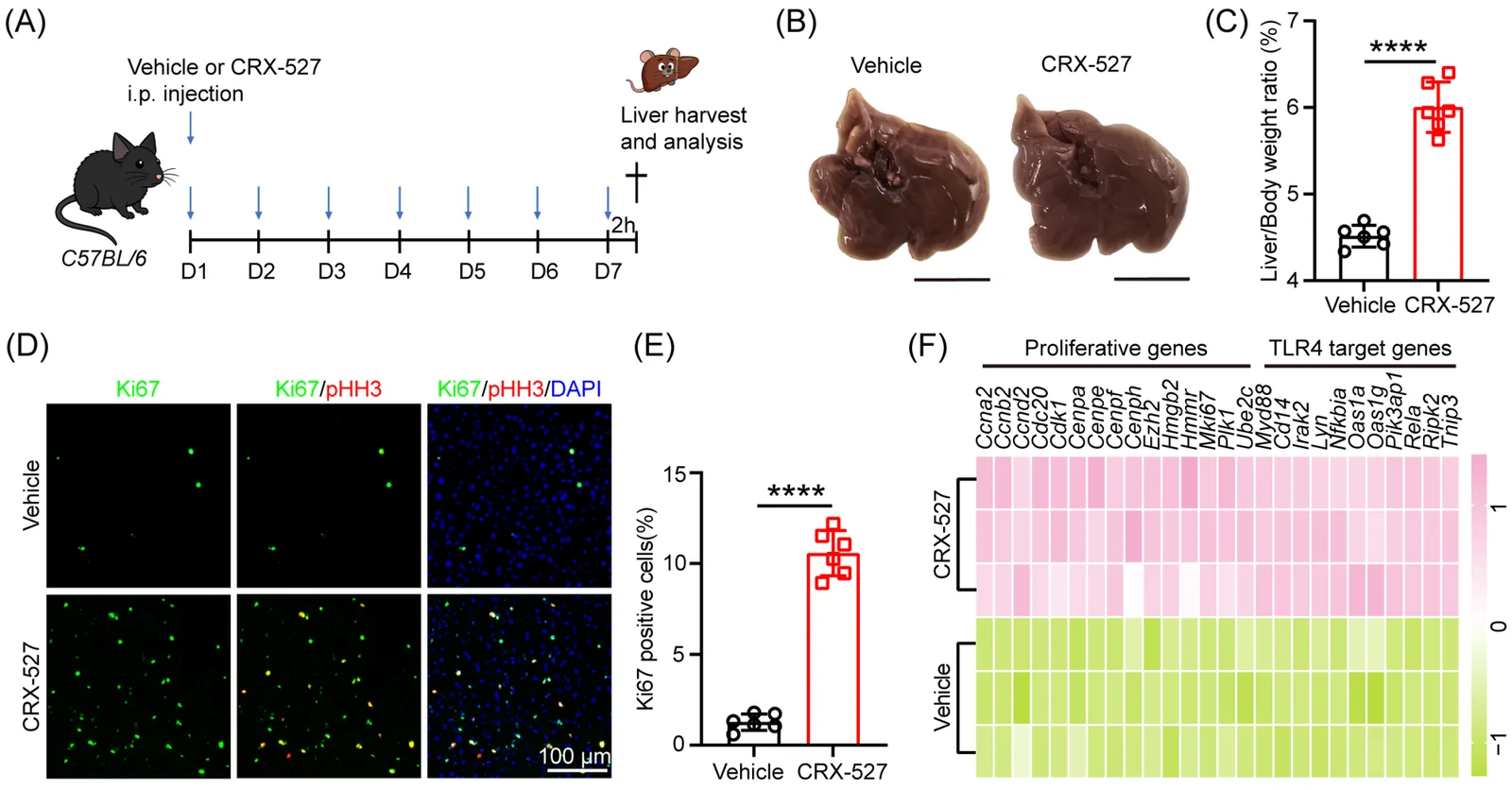
CRX-527 promotes the proliferation of hepatocytes in wild-type mice. (**A**) Experimental schematic. Wild-type C57BL/6 mice received daily intraperitoneal injections of CRX-527 (0.25 mg/kg) or vehicle for 7 consecutive days. (**B**) Representative liver photos from vehicle-treated or CRX-527-treated mice. Scale bar: 1 cm. (**C**) Liver-to-body weight ratio in mice treated as described in (**A**) (*n* = 6 per group). (**D**) Representative immunofluorescence images showing Ki67 (green), pHH3 (red) and DAPI (blue) staining in liver sections from mice treated as described in (**A**). (**E**) Quantification of the percentage of Ki67-positive cells (Ki67/DAPI) in liver sections from mice treated as described in (**A**) (*n* = 6 per group). (**F**) Differentially expressed proliferative genes and TLR4 target genes in the livers of vehicle-treated or CRX-527-treated mice (*n* = 3 per group). Unpaired two-tailed *t*-test (**C**,**E**). Data are represented as means ± SD; **** *p* < 0.0001.

**Figure 2:**
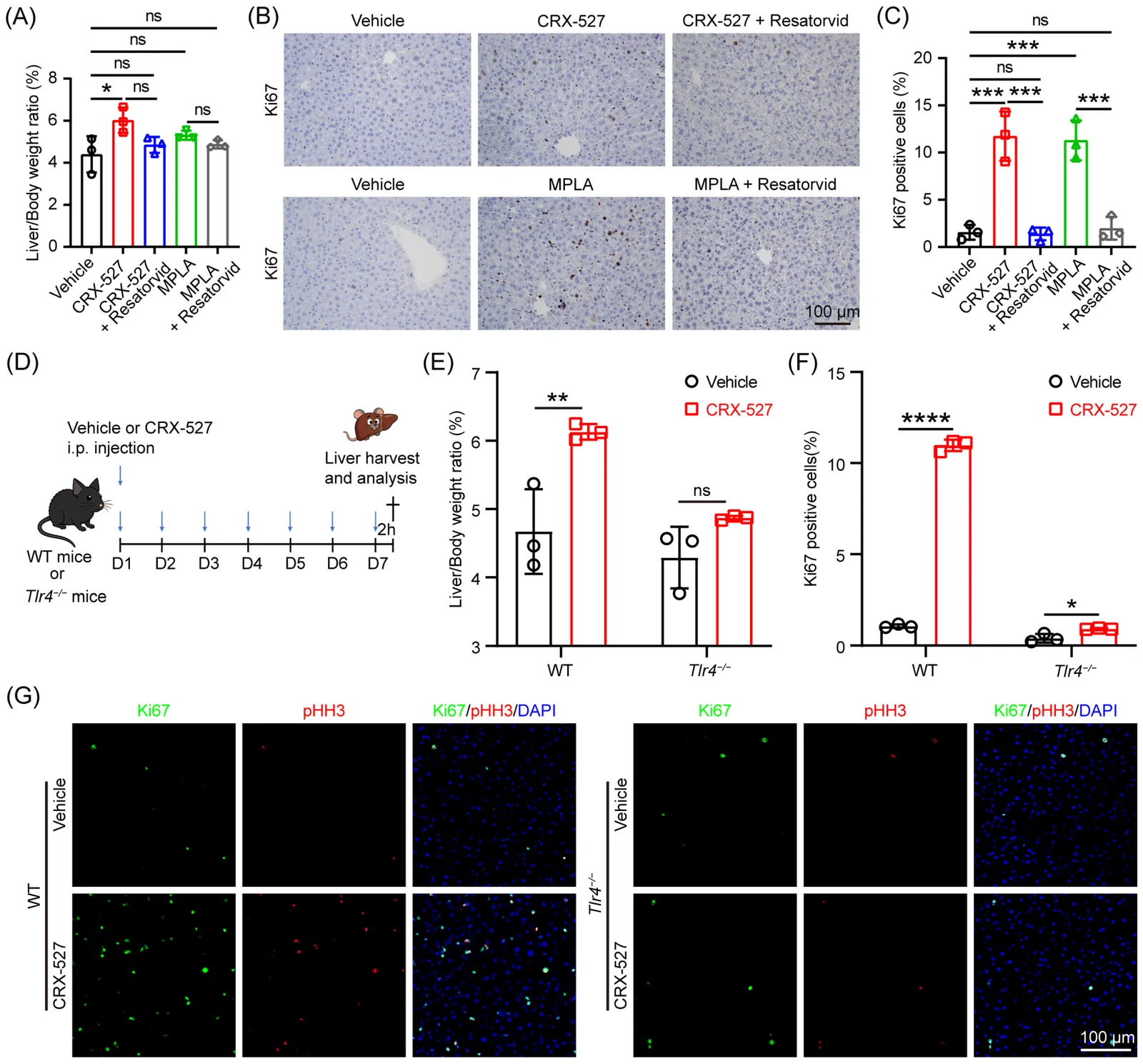
CRX-527 promotes hepatocyte proliferation via TLR4. (**A**) Liver to body weight ratio of mice treated with vehicle, CRX-527 alone, CRX-527 plus resatorvid, MPLA (0.5 mg/kg) alone, or MPLA plus resatorvid (*n* = 3 per group). (**B**) Representative images of Ki67 staining on liver sections from mice as described in (**A**). (**C**) Quantification of the percentage of Ki67/DAPI staining conducted on liver sections from mice as described in (**A**) (*n* = 3 per group). (**D**) Experimental schematic. Wild-type mice and *Tlr4^−/−^* mice were treated with vehicle or CRX-527 for 7 days. (**E**) Liver to body weight ratio of mice treated as described in (**D**) (*n* = 3 per group). (**F**) Quantification of the percentage of Ki67^+^ cells conducted on liver sections from mice as described in (**D**) (*n* = 3 per group). (**G**) Representative immunofluorescence staining of Ki67 (green), pHH3 (red) and DAPI (blue) staining on liver sections from mice as described in (**D**). One-way ANOVA with Tukey’s correction (**A**,**C**); two-way ANOVA with Sidak’s correction (**E**,**F**). Data are represented as means ± SD; * *p* < 0.05, ** *p* < 0.01, *** *p* < 0.001, **** *p* < 0.0001, ns, not significant.

**Figure 3:**
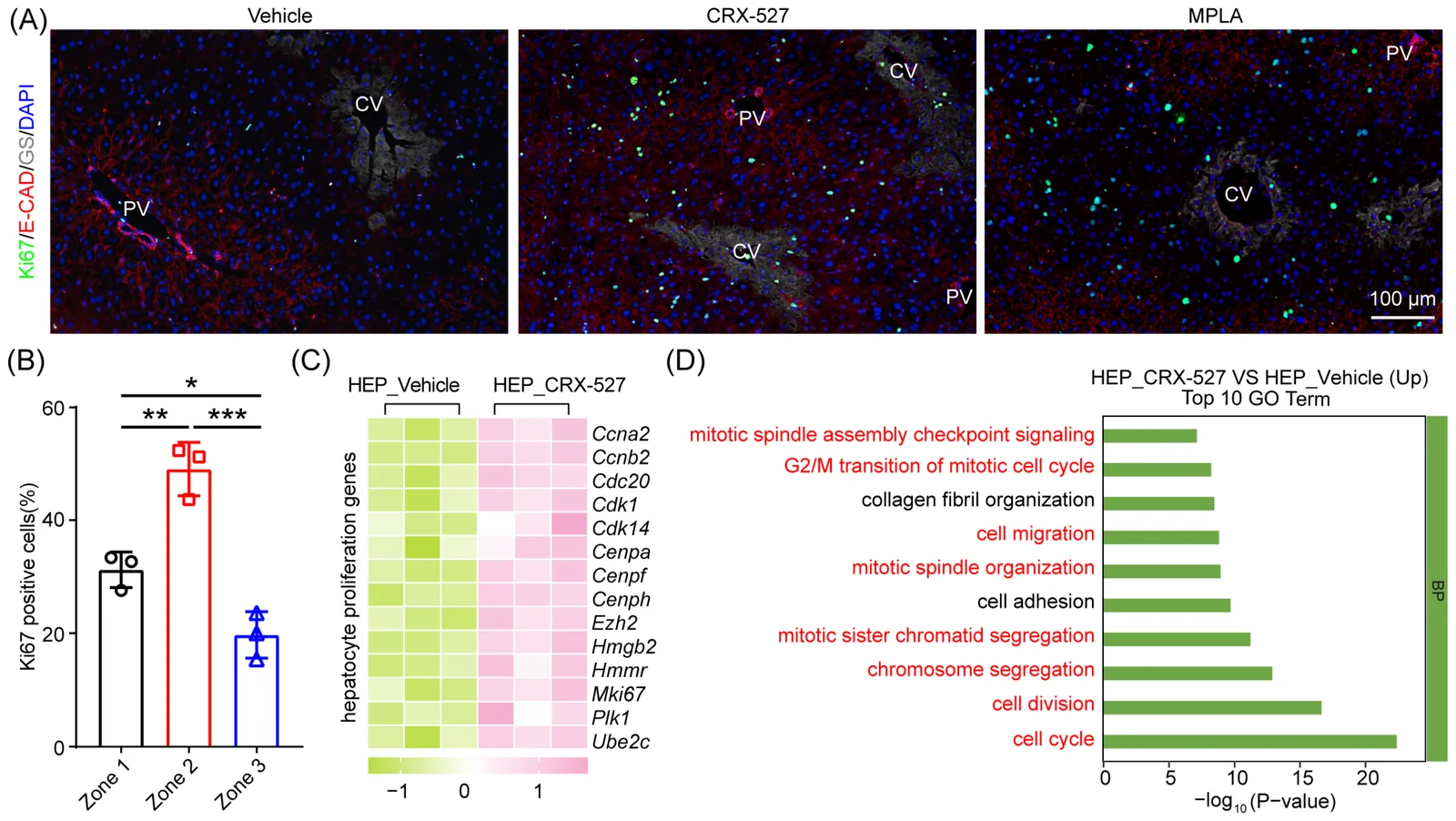
CRX-527 induces zone 2 hepatocyte proliferation and activates cell cycle programs. (**A**) Representative immunofluorescence staining of Ki67, zone 1 marker E-cadherin (E-CAD) and zone 3 marker glutamine synthetase (GS) on liver sections from mice treated with vehicle, CRX-527, or MPLA. (**B**) Proportions of Ki67-positive hepatocytes in hepatic zone 1 (E-CAD^+^), zone 2 (E-CAD^−^/GS^−^), and zone 3 (GS^+^) in liver sections from mice treated with CRX-527 (*n* = 3 per group). (**C**) Heatmap of differentially expressed proliferation-associated genes in hepatocytes from vehicle- and CRX-527-treated mice (*n* = 3 per group). (**D**) Gene ontology (GO) analysis of biological pathways revealed an enrichment of cell proliferation-associated signaling pathways in hepatocytes from vehicle-treated and CRX-527-treated mice. One-way ANOVA with Tukey’s correction (**B**). Data are represented as means ± SD; * *p* < 0.05; ** *p* < 0.01; *** *p* < 0.001.

**Figure 4:**
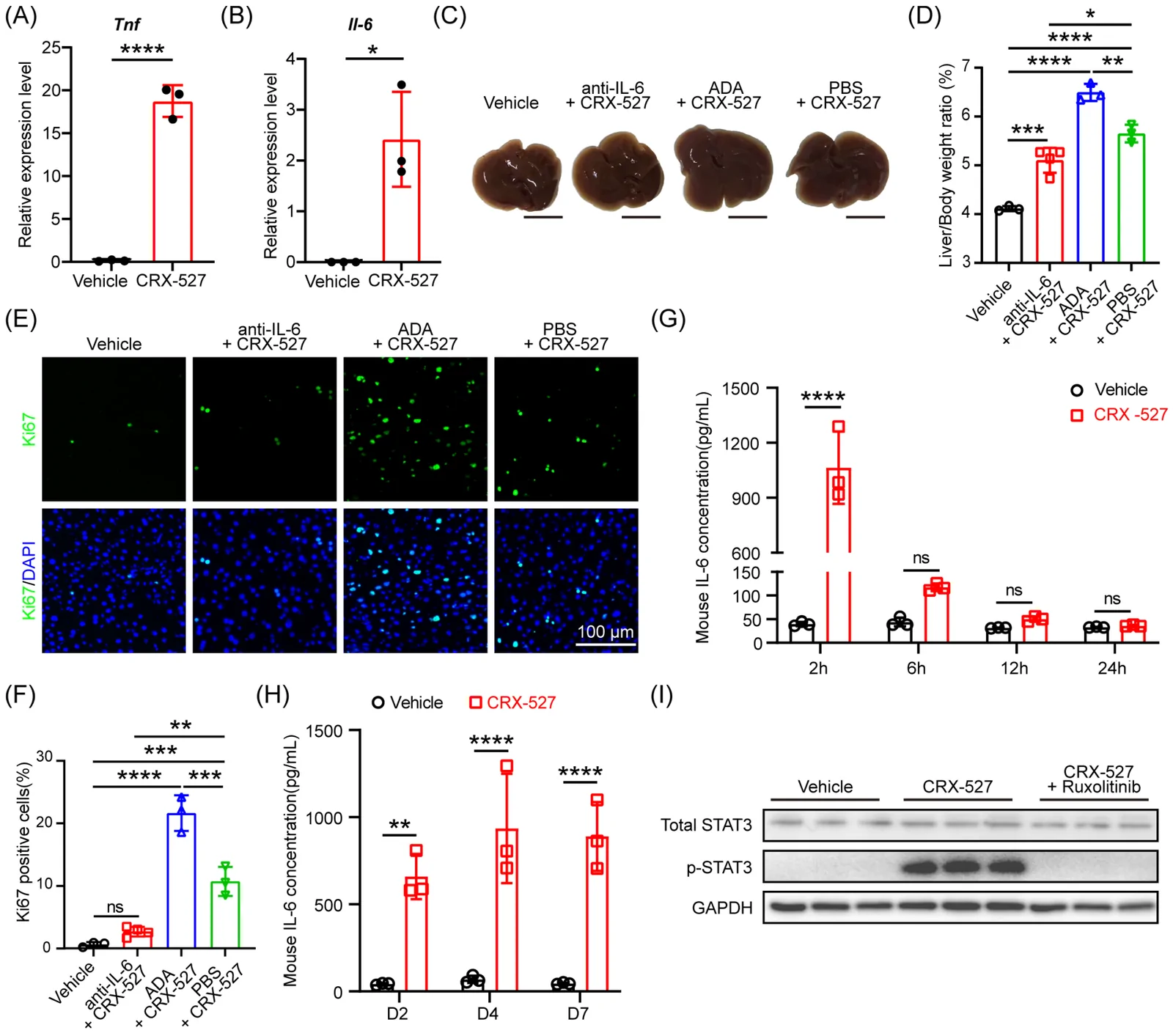
CRX-527 promotes hepatocyte proliferation in an IL-6-dependent manner. (**A**,**B**) Hepatic mRNA expression levels of the pro-inflammatory cytokines *Tnf* (**A**) and *Il-6* (**B**) in the livers of vehicle-treated or CRX-527-treated mice analyzed by bulk RNA-seq. (**C**) Representative liver photos from mice treated with vehicle, CRX-527 alone, CRX-527 plus anti-IL-6 antibody, or CRX-527 plus adalimumab (ADA). Scale bars: 1 cm. (**D**) Liver-to-body weight ratio in mice treated as described in (**C**) (*n* = 3 in vehicle-treated, ADA+CRX-527-treated, and PBS+CRX-527-treated group; *n* = 4 for anti-IL6+CRX-527 treated group). (**E**) Representative immunofluorescence staining of Ki67 (green) and DAPI (blue) on liver sections from mice treated as described in (**C**). Ki67/DAPI staining for vehicle, CRX-527 plus anti-IL-6, CRX-527 plus AD(A) or CRX-527 alone. (**F**) Quantification of the percentage of Ki67-positive cells (Ki67/DAPI) in liver sections from mice as described in (**C**) (*n* = 3 for vehicle-treated, ADA+CRX-527-treated, and PBS+CRX-527-treated groups; *n* = 4 for anti-IL-6+CRX-527-treated group). (**G**) Serum IL-6 levels in vehicle-treated and CRX-527-treated mice at 2, 6, 12 and 24 h after injection (*n* = 3 per time point). (**H**) Serum IL-6 levels at 2 h post-administration on days 2, 4, and 7 in wild-type C57BL/6 mice treated with vehicle or CRX-527 (*n* = 3 per time point). (**I**) Representative Western blot images showing protein levels of total STAT3, p-STAT3 (Tyr705), and GAPDH in liver tissues from mice treated with vehicle, CRX-527 alone, or CRX-527 plus JAK1/2 inhibitor ruxolitinib. Unpaired two-tailed *t*-test (**A**,**B**); one-way ANOVA with Tukey’s correction (**D**,**F**); two-way ANOVA with Sidak’s correction (**G**,**H**). Data are presented as means ± SD; * *p* < 0.05; ** *p* < 0.01; *** *p* < 0.001; **** *p* < 0.0001; ns, not significant. (The original Western blot images were supplied in Supplementary Figure S4).

**Figure 5:**
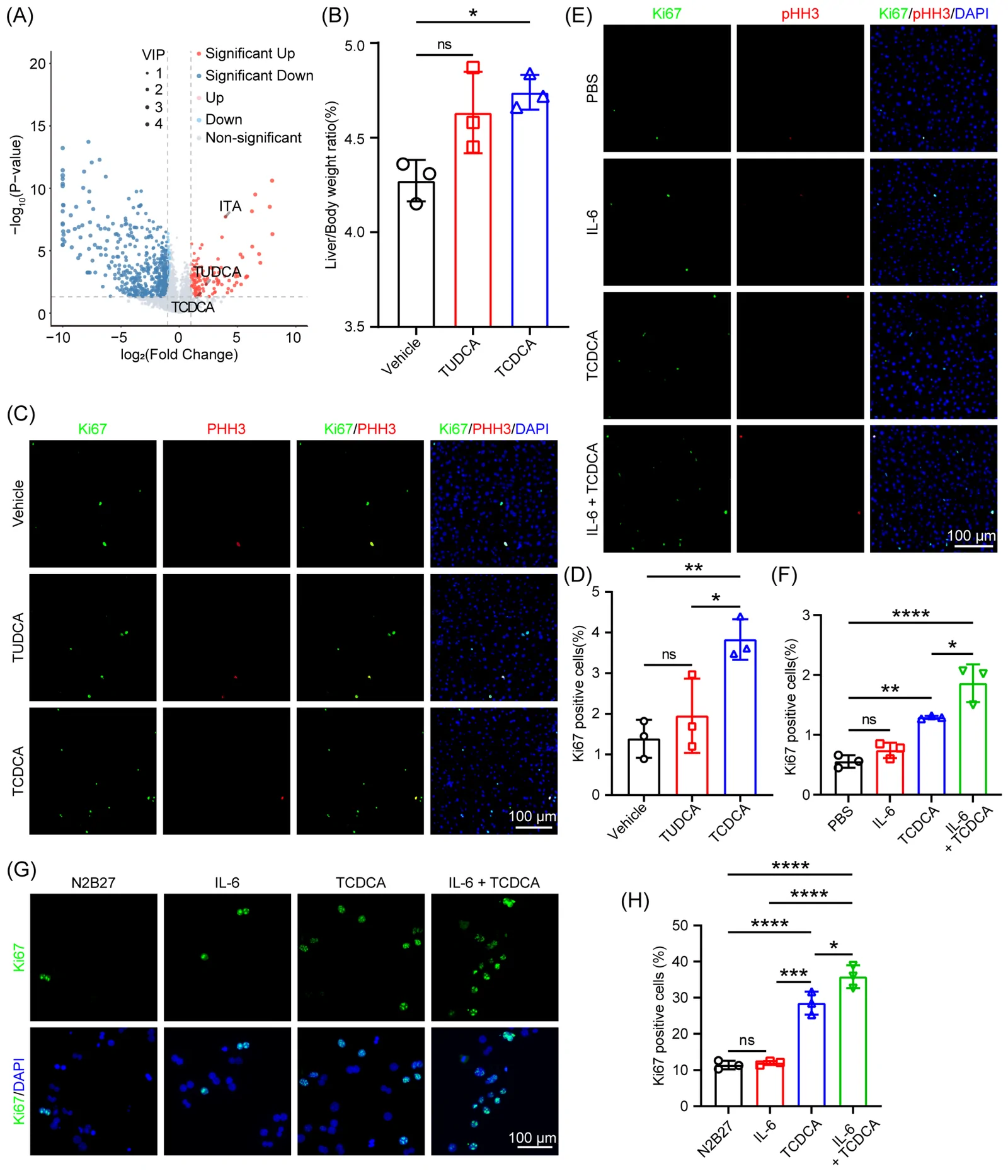
IL-6 and TCDCA have a combined effect in promoting hepatocyte proliferation. (**A**) Volcano plot showing differential serum metabolites between vehicle- and CRX-527-treated mice (*n* = 6 per group). (**B**) Liver-to-body weight ratio in mice treated with vehicle, TUDCA (25 mg/kg), or TCDCA (25 mg/kg) (*n* = 3 per group). (**C**) Representative immunofluorescence staining of Ki67 (green), pHH3 (red), and DAPI (blue) on liver sections from mice treated as described in (**B**). Ki67/DAPI staining is shown for vehicle (**top**), TUDCA (25 mg/kg, **middle**), and TCDCA (25 mg/kg, **bottom**). (**D**) Quantification of the percentage of Ki67-positive cells (Ki67/DAPI) in liver sections from mice treated with PBS, TUDCA, TCDCA (*n* = 3 per group). (**E**) Representative immunofluorescence staining of Ki67 (green), pHH3 (red), and DAPI (blue) on liver sections from mice treated with PBS, IL-6, TCDCA or IL-6 plus TCDCA. (**F**) Quantification of the percentage of Ki67-positive cells (Ki67/DAPI) in liver sections from mice treated with PBS, IL-6, TCDCA or IL-6 plus TCDCA (*n* = 3 per group). (**G**) Representative immunofluorescence staining of Ki67 (red) and DAPI (blue) in primary hepatocytes from C57BL/6 WT mice after 48 h of in vitro culture with N2B27 medium, IL-6 (20 ng/mL), TCDCA (100 μM), or IL-6 plus TCDCA. (**H**) Quantification of the percentage of Ki67-positive cells (Ki67/DAPI) in primary hepatocytes from wild-type C57BL/6 mice after 48 h of culture in N2B27, IL-6, TCDCA or IL-6 plus TCDCA (*n* = 3 mice, for each mouse, three random microscopic fields were quantified and averaged prior to statistical analysis.). One-way ANOVA with Tukey’s correction (**B**,**D**,**F**,**H**). Data are presented as means ± SD; * *p* < 0.05; ** *p* < 0.01; *** *p* < 0.001; **** *p* < 0.0001; ns, not significant.

**Figure 6:**
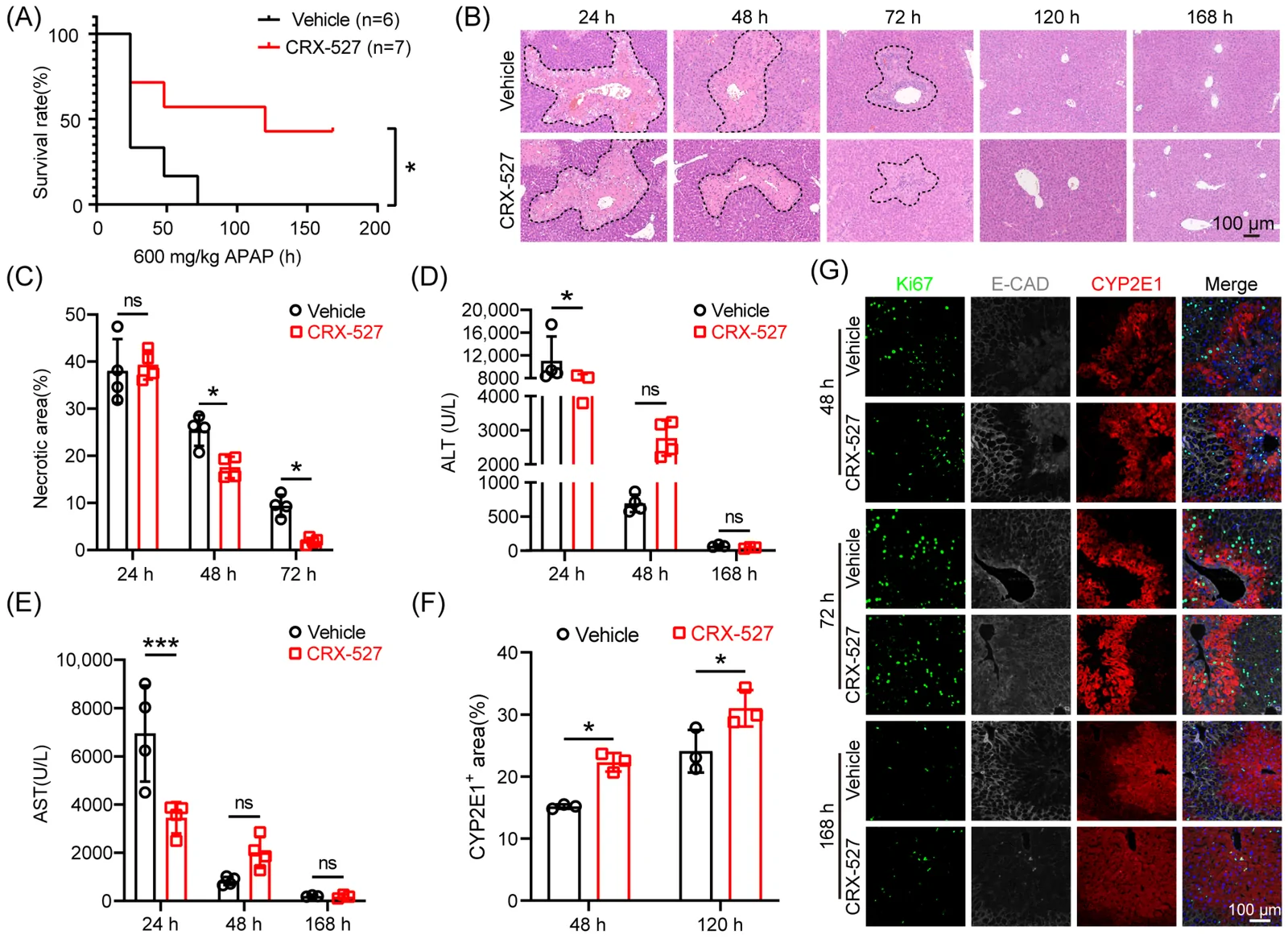
CRX-527 attenuates APAP-induced liver injury. (**A**) Kaplan–Meier survival curves of mice following a lethal dose of APAP (600 mg/kg) with or without CRX-527 treatment. (**B**) Representative H&E staining of liver sections from mice treated with vehicle or CRX-527 during 400 mg/kg APAP-induced liver injury. (**C**) Quantification of the necrotic area in liver sections from mice as described in (**B**) (*n* = 4 per group). (**D**,**E**) Serum ALT (**D**) and AST (**E**) levels in vehicle- and CRX-527-treated mice following 400 mg/kg APAP challenge (*n* = 4 per group at 24, 48 and 72 h; *n* = 3 per group at 120 and 168 h). (**F**) Quantification of the CYP2E1-positive area in liver sections from vehicle- and CRX-527-treated mice following 400 mg/kg APAP-induced liver injury (*n* = 3 per group). (**G**) Representative immunofluorescence staining of Ki67 (green), E-CAD (gray), CYP2E1 (red), and DAPI (blue) on liver sections following 400 mg/kg APAP challenge. Two-way ANOVA with Sidak’s correction (**C**–**F**). Data are represented as means ± SD; * *p* < 0.05; *** *p* < 0.001; ns, not significant.

**Figure 7:**
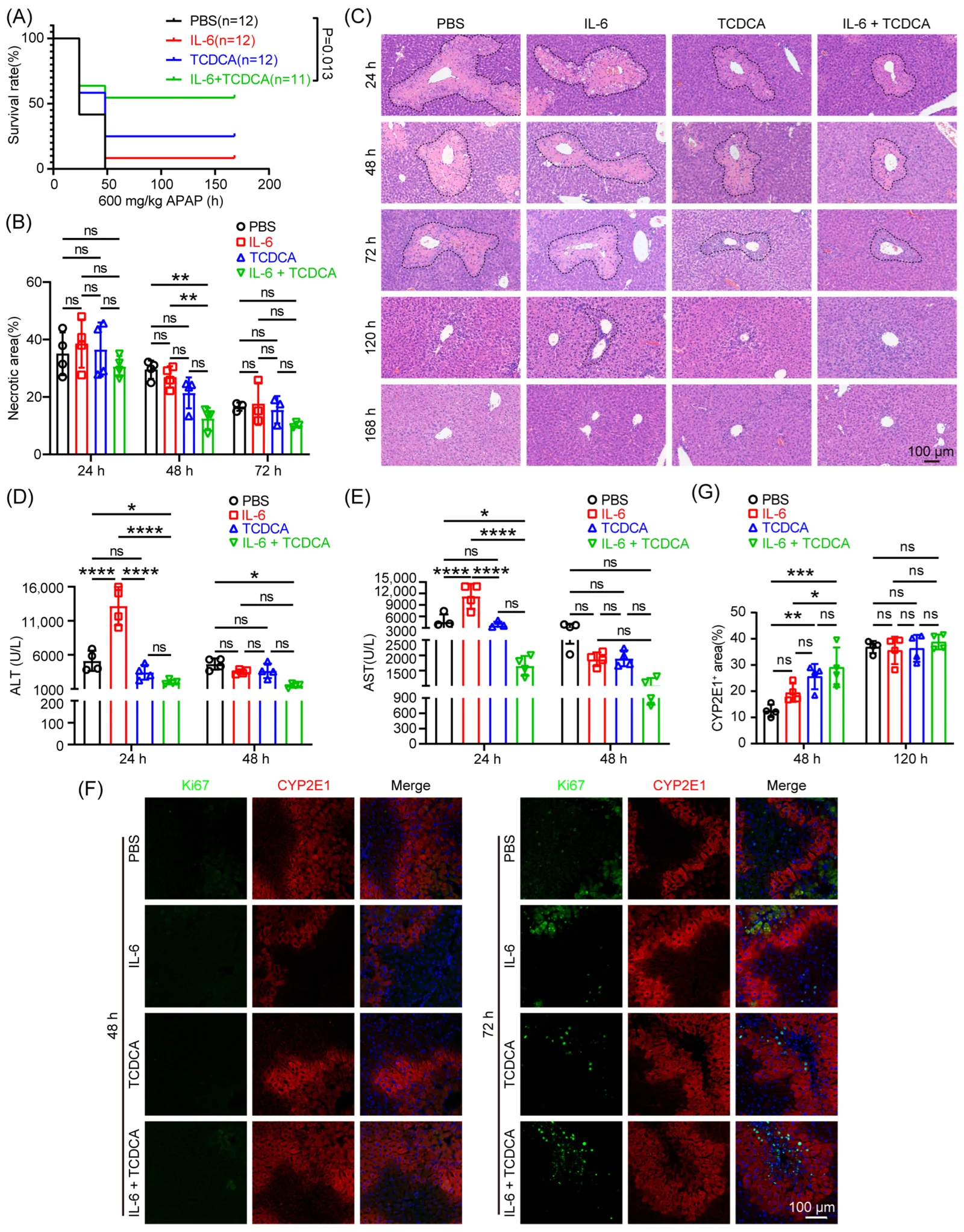
Combined IL-6 and TCDCA treatment attenuate APAP-induced liver injury. (**A**) Kaplan-Meier survival curves of mice following a lethal dose of APAP (600 mg/kg) treated with PBS, IL-6, TCDCA or IL-6 plus TCDCA. (**B**) Quantification of the necrotic area in liver sections from mice treated with PBS, IL-6, TCDCA or IL-6 plus TCDCA during 400 mg/kg APAP-induced liver injury (*n* = 4 per group at 24 and 48 h; *n* = 3 per group at 72 h). (**C**) Representative H&E-staining of liver sections from mice treated as described in (**B**). (**D**,**E**) Serum ALT (**D**) and AST (**E**) levels in the indicated treatment groups (*n* = 4 per group) following 400 mg/kg APAP challenge. (**F**) Representative immunofluorescence staining of Ki67 (green), E-CAD (gray), CYP2E1 (red), and DAPI (blue) on liver sections from mice treated with PBS, IL-6, TCDCA or IL-6 plus TCDCA during 400 mg/kg APAP-induced liver injury. (**G**) Quantification of the CYP2E1-positive area in liver sections from mice treated with PBS, IL-6, TCDCA or IL-6 plus TCDCA during 400 mg/kg APAP-induced liver injury (*n* = 4 per group). Two-way ANOVA with Sidak’s correction (**B**,**D**,**E**,**G**). Data are presented as means ± SD; * *p* < 0.05; ** *p* < 0.01; *** *p* < 0. 001; **** *p* < 0.0001; ns, not significant.

## Data Availability

The raw sequence data reported in this paper have been deposited in the Genome Sequence Archive (Genomics, Proteomics & Bioinformatics 2025) in National Genomics Data Center (Nucleic Acids Res 2025), China National Center for Bioinformation/Beijing Institute of Genomics, Chinese Academy of Sciences (GSA: CRA048331) that are publicly accessible at https://ngdc.cncb.ac.cn/gsa, accessed on 22 September 2026. The metabolomics data are publicly available in the OMIX repository under the accession number OMIX019562. All biochemical data and histopathological records are securely stored and can be shared for academic and non-commercial research purposes.

## Supplementary Materials

The following supporting information can be downloaded at: https://www.mdpi.com/article/10.3390/biology15191725/s1, Figure S1. CRX-527 treatment does not cause overt liver injury. (A) Representative hematoxylin and eosin (H&E)-stained liver sections from mice treated with vehicle or CRX-527. (B–E) Serum level of alanine aminotransferase (ALT, B), aspartate transaminase in (AST, C), total bilirubin (T-BIL, D), and alkaline phosphatase (ALP, E) in vehicle- and CRX-527-treated mice (*n* = 3 per group). Data are represented as means ± SD; * *p* < 0.05; ns, not significant; Figure S2. MPLA treatment does not cause overt liver injury. (A) Representative H&E-stained in liver sections from mice treated with vehicle or MPLA. (B-E) Serum levels of ALT (B), AST (C), T-BIL (D), and ALP (E) in vehicle- and MPLA-treated mice (*n* = 3 per group). Data are represented as means ± SD; *** *p* < 0.001; ns, not significant; Figure S3. CRX-527 promotes hepatocyte proliferation. (A) Representative immunofluorescence images showing Ki67 (green), HNF4A (red), and DAPI (blue) in liver sections from vehicle-, CRX-527-, or MPLA-treated mice. (B) Proportion of Ki67^+^HNF4A^+^ double-positive cells among total Ki67-positive cells in liver sections from vehicle-, CRX-527-, or MPLA- treated mice (*n* = 3 per group). (C) Representative immunofluorescence images showing Ki67 (green), ALB (red), and DAPI (blue) in liver sections from vehicle-, CRX-527-, or MPLA- treated mice. Data are represented as means ± SD; * *p* < 0.05; **** *p* < 0.0001; Figure S4. The original Western blot images for Figure 4I. Figure S5. Inhibition of IL-6/JAK2/STAT3 signaling attenuates CRX-527-induced hepatocyte proliferation. (A) Liver-to-body weight ratio in mice treated with vehicle, CRX-527 alone, or CRX-527 plus ruxolitinib (*n* = 3 for vehicle- and CRX-527-treated groups; *n* = 7 for CRX-527 plus ruxolitinib group). (B) Representative immunofluorescence images showing Ki67 (green) and DAPI (blue) in liver sections from mice treated as described in (A). (C) Quantification of the percentage of Ki67-positive cells (Ki67/DAPI) in liver sections from mice treated with vehicle, CRX-527 alone, or CRX-527 plus ruxolitinib (*n* = 3 for vehicle- and CRX-527-treated groups; *n* = 7 for CRX-527 plus ruxolitinib group). Data are presented as means ± SD; * *p* < 0.05; **** *p* < 0.0001; ns, not significant; Figure S6. LC–MS/MS-based untargeted metabolomic profiling of serum from CRX-527- and vehicle-treated mice. (A) Principal component analysis (PCA) plot of serum metabolite profiles from vehicle- and CRX-527-treated mice (*n* = 6 per group). (B) Pie chart showing the distribution of detected serum metabolites by chemical class. (C) Bar plot showing the number of detected metabolites at each annotation confidence level. (D) Numbers of significantly upregulated and downregulated serum metabolites between vehicle- and CRX-527-treated mice. (E) Quantification of the percentage of Ki67-positive cells (Ki67/DAPI) in liver sections from mice treated with vehicle or 4-octyl itaconate (4-OI) (*n* = 3 for vehicle-treated group; *n* = 5 for 4-OI-treated group). (F) Representative immunofluorescence images showing Ki67 (green) and DAPI (blue) in liver sections from vehicle- and 4-OI-treated mice. Data are presented as means ± SD; ns, not significant.

## Abbreviations

APAP	Acetaminophen
TCDCA	Taurochenodeoxycholic acid
TLR4	Toll-like receptor 4
DILI	Drug-induced liver injury
NAPQI	N-acetyl-p-benzoquinone imine
ALT	Alanine aminotransferase
AST	Aspartate transaminase
T-BIL	Total bilirubin
ALP	Alkaline phosphatase
MPLA	Monophosphoryl lipid A
GS	Glutamine synthetase
RNA-seq	RNA-sequencing
GO	Gene Ontology
ADA	Adalimumab
ITA	Itaconic acid
TUDCA	Tauroursodeoxycholic acid
4-OI	4-octyl itaconate
