# Supplementary material for: Pharmacological TLR4 Activation Promotes Hepatocyte Proliferation and Liver Repair Through Coordinated IL-6 and TCDCA Signaling
Source: Biology (Basel). 2026 Oct 1;15(19):1725. doi: 10.3390/biology15191725 (PMC13663612; doi:10.3390/biology15191725)
Supplement: Supplementary file 1 [file biology-15-01725-s001.zip › biology-4526870-supplementary.pdf]

Supplementary Figures

Supplementary Figure S1

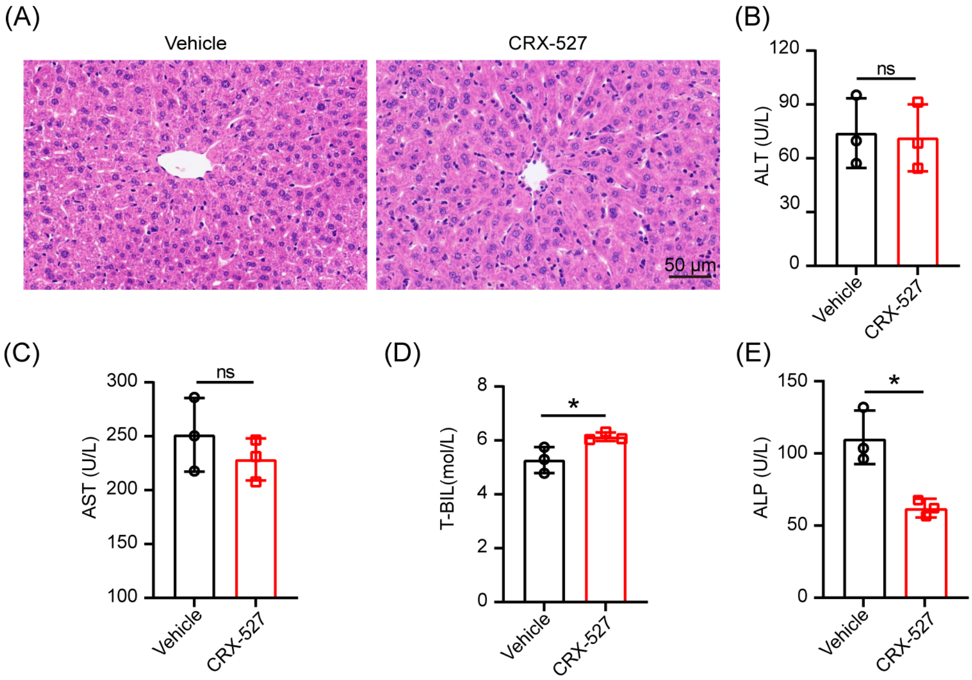

Supplementary Figure S1. CRX-527 treatment does not cause overt liver injury. (A) Representative hematoxylin and eosin (H&E)-stained liver sections from mice treated with vehicle or CRX-527. (B-E) Serum level of alanine aminotransferase (ALT, B), aspartate transaminase in (AST, C), total bilirubin (T-BIL, D), and alkaline phosphatase (ALP, E) in vehicle- and CRX-527-treated mice (n = 3 per group). Data are represented as means ± SD; * $p < 0.05$; ns, not significant.

Supplementary Figure S2

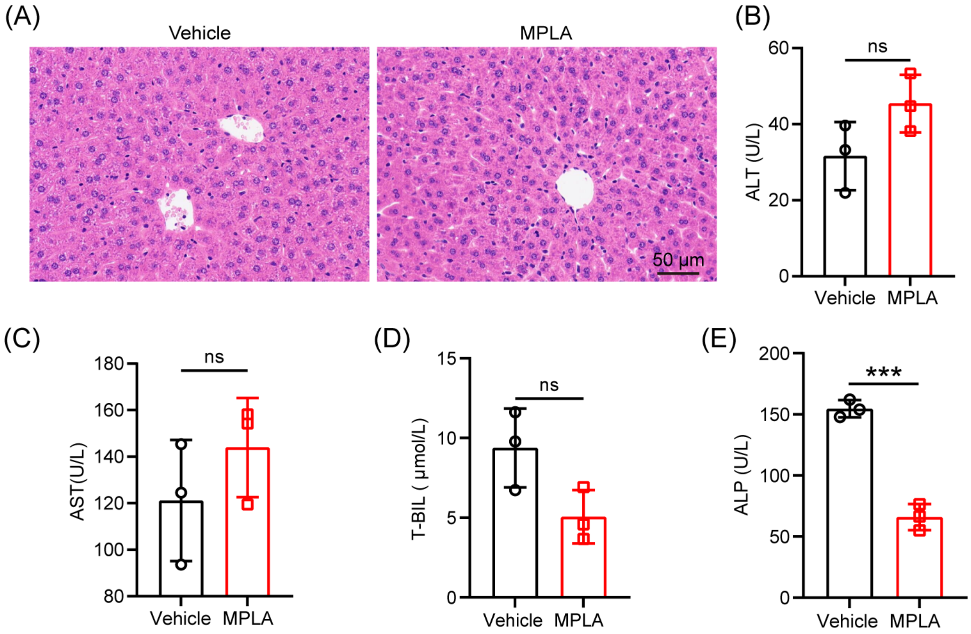

Supplementary Figure S2. MPLA treatment does not cause overt liver injury. (A) Representative H&E-stained in liver sections from mice treated with vehicle or MPLA. (B-E) Serum levels of ALT (B), AST (C), T-BIL (D), and ALP (E) in vehicle- and MPLA-treated mice (*n* = 3 per group). Data are represented as means ± SD; *** $p < 0.001$; ns, not significant.

Supplementary Figure S3

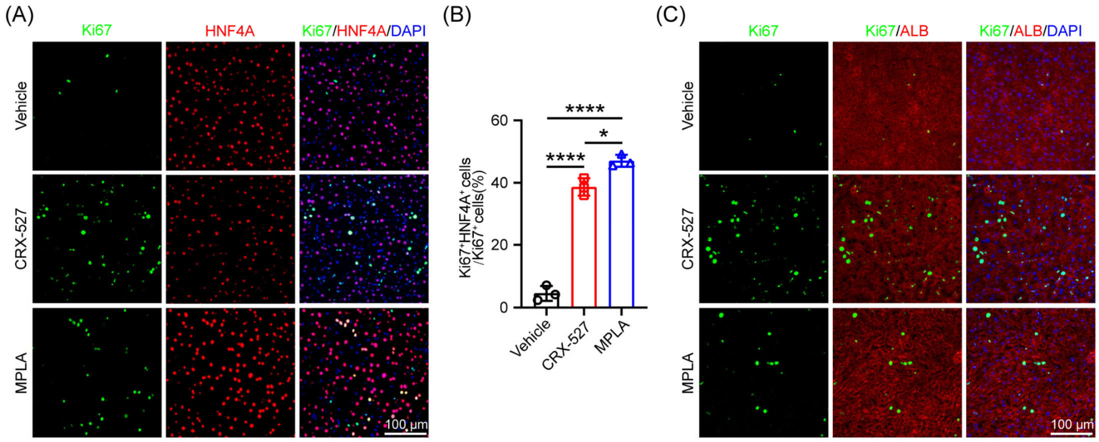

Supplementary Figure S3. CRX-527 promotes hepatocyte proliferation. (A) Representative immunofluorescence images showing Ki67 (green), HNF4A(red), and DAPI (blue) in liver sections from vehicle-, CRX-527-, or MPLA-treated mice. (B) Proportion of $Ki67^{+}HNF4A^{+}$ double-positive cells among total Ki67-positive cells in liver sections from vehicle-, CRX-527-, or MPLA- treated mice ($n = 3$ per group). (C) Representative immunofluorescence images showing Ki67 (green), ALB (red), and DAPI (blue) in liver sections from vehicle-, CRX-527-, or MPLA- treated mice. Data are represented as means ± SD; * $p < 0.05$; **** $p < 0.0001$.

Supplementary Figure S4

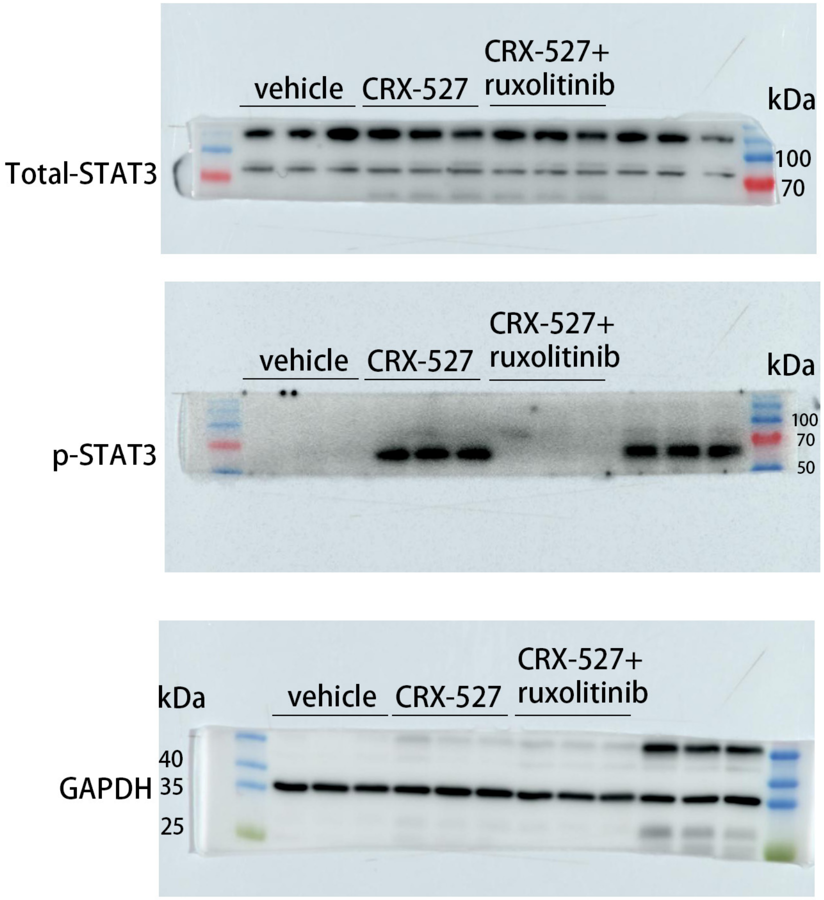

Supplementary Figure S4. The original Western blot images for figure 4I.

Supplementary Figure S5

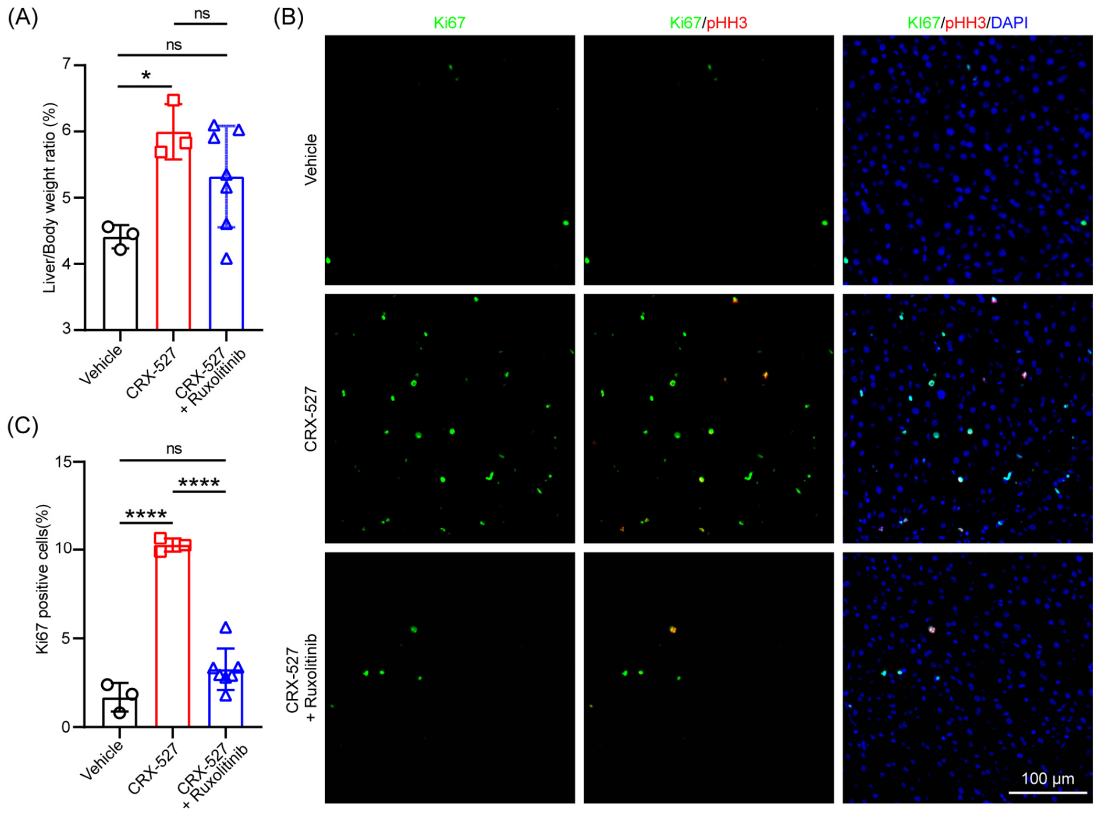

Supplementary Figure S5. Inhibition of IL-6/JAK2/STAT3 signaling attenuates CRX-527-induced hepatocyte proliferation. (A) Liver-to-body weight ratio in mice treated with vehicle, CRX-527 alone, or CRX-527 plus ruxolitinib ($n = 3$ for vehicle- and CRX-527-treated groups; $n = 7$ for CRX-527 plus ruxolitinib group). (B) Representative immunofluorescence images showing Ki67 (green) and DAPI (blue) in liver sections from mice treated as described in (A). (C) Quantification of the percentage of Ki67-positive cells (Ki67/DAPI) in liver sections from mice treated with vehicle, CRX-527 alone, or CRX-527 plus ruxolitinib ($n = 3$ for vehicle- and CRX-527-treated groups; $n = 7$ for CRX-527 plus ruxolitinib group). Data are presented as means ± SD; * $p < 0.05$; **** $p < 0.0001$; ns, not significant.

Supplementary Figure S6

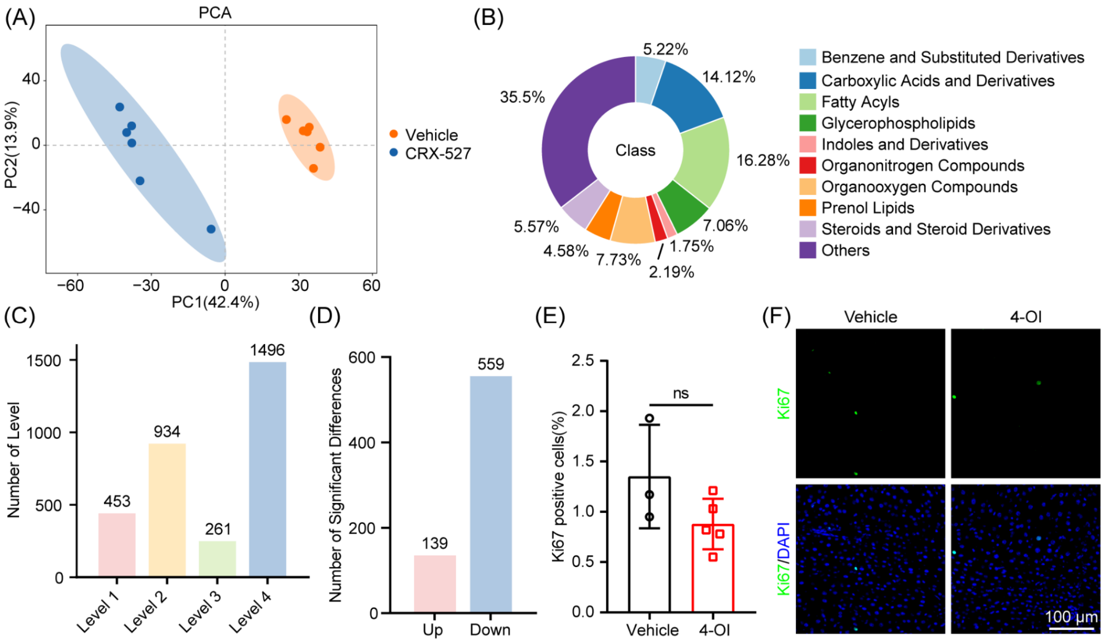

Supplementary Figure S6. LC–MS/MS-based untargeted metabolomic profiling of serum from CRX-527- and vehicle-treated mice. (A) Principal component analysis (PCA) plot of serum metabolite profiles from vehicle- and CRX-527-treated mice (n = 6 per group).(B) Pie chart showing the distribution of detected serum metabolites by chemical class.(C) Bar plot showing the number of detected metabolites at each annotation confidence level.(D) Numbers of significantly upregulated and downregulated serum metabolites between vehicle- and CRX-527-treated mice.(E) Quantification of the percentage of Ki67-positive cells (Ki67/DAPI) in liver sections from mice treated with vehicle or 4-octyl itaconate (4-OI) (n = 3 for vehicle-treated group; n = 5 for 4-OI-treated group).(F) Representative immunofluorescence images showing Ki67 (green) and DAPI (blue) in liver sections from vehicle- and 4-OI-treated mice. Data are presented as means ± SD; ns, not significant.
